# Herlyn Werner Wunderlich Syndrome with Hydrocolpos: A Case Report

**DOI:** 10.31729/jnma.8520

**Published:** 2024-03-31

**Authors:** Saurav Sen Oli, Shova Sapkota, Rupa Bajagain, Rachana Saha, Suman Paudel

**Affiliations:** 1 Kathmandu Medical College and Teaching Hospital, Sinamangal, Kathmandu, Nepal; 2 Department of Obstetrics and Gynaecology, Kathmandu Medical College and Teaching Hospital, Sinamangal, Kathmandu, Nepal; 3 Department of Radiology, Kathmandu Medical College and Teaching Hospital, Sinamangal, Kathmandu, Nepal

**Keywords:** *case reports*, *hydrocolpos*, *mullerian ducts*, *uterine didelphys*

## Abstract

Herlyn-Werner-Wunderlich Syndrome is a very rare congenital malformation of the urogenital tract involving both the Mullerian and Wolffian ducts characterized by the triad uterine diadelphys, obstructed vagina, and unilateral renal agenesis. If not diagnosed on time it may progress to adverse gynecological complications making timely diagnosis and treatment crucial. We hereby present a 14-year girl with right flank pain diagnosed as Herlyn-Werner-Wunderlich Syndrome by ultrasound scan which was managed surgically with drainage of hydrocolpos and marsupialization of vaginal septum. On two weeks follow up patient had symptomatic improvement with no any complications.

## INTRODUCTION

Herlyn-Werner-Wunderlich Syndrome (HWWS) is a rare congenital anomaly with the incidence of 1 in 1000,000 girls.^1^ It is caused by anomaly of wolffian duct and a failed fusion of two mullerian ducts resulting into triad of uterine didelphys, obstructed hemivagina and ipsilateral renal agenesis.^2,3^ Patients usually present in the teenage years, shortly after menarche with progressive cyclic pelvic pain, dysmenorrhea, pelvic mass and if not detected early, may present with complications like ascending infection, pyocolpos and endometriosis.^4,5^ Ultrasound scan of abdomen and pelvis can be used as early investigation followed by magnetic resonance imaging (MRI) while laparoscopy being gold standard for diagnosis.^3^ Surgical management as vaginal septoplasty is the treatment of choice.^4^ We present a case of HWWS in a 14 year old girl who had not attained menarche and had presented with right flank pain.

## CASE REPORT

A 14-year female presented to our center with complaints of right flank pain for 4 days which was acute on onset, dull aching, non-radiating, exaggerated movement and severe enough to restrict her daily activities. She gave no history of burning micturition, fever, hematuria, nausea, vomiting and her bladder and bowel habit were as usual. She had not attained menarche till the time of presentation. On clinical examination, her vitals were within the normal limit but per abdominal examination revealed suprapubic tenderness.

**Figure 1:**
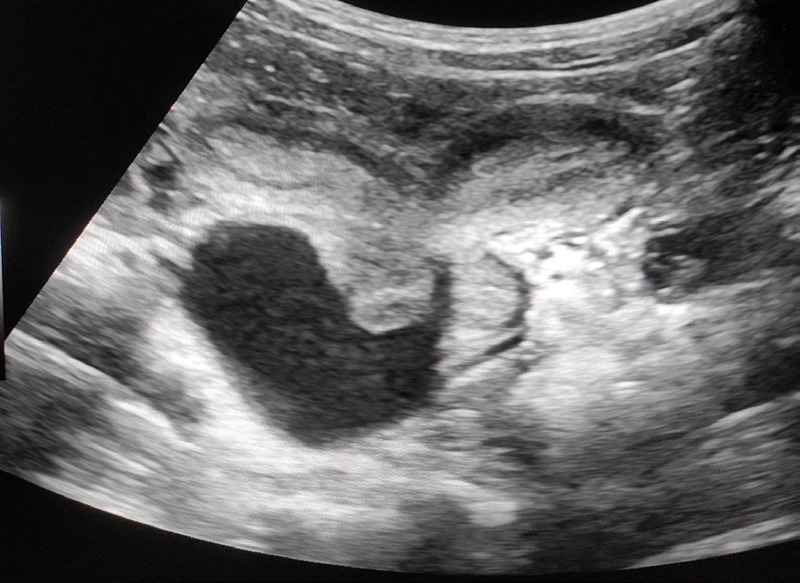
Uterine didelphys with fluid collection in the right hemivagina.

On further investigation, her baseline investigations including complete blood count, renal function test, urine routine microscopic examination were within normal limits. Further ultrasound (USG) of abdomen and pelvis was done to rule out surgical causes of acute abdomen which showed absent kidney on the right side with 109 mm left kidney, two uterine bodies with a separate cervix and vaginal canal suggesting uterine didelphys. A well-defined fluid collection measuring approximately 65 x 47 x 52 mm (84 cc) was noted in the right vaginal canal likely representing hematocolpos. Thickness of endometrium on right and left uterine cavity measured approximately 7.8 mm and 7.1 mm respectively. All these USG findings of uterine didelphys with obstructed right hemivagina and right renal agenesis suggested the diagnosis of Herlyn-Werner-Wunderlich syndrome (Figure 1).

**Figure 2:**
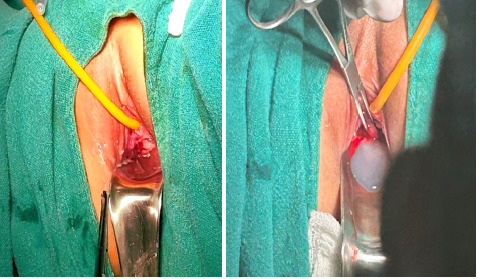
A) Right imperforated hemivagina bulged out towards introitus, B) Clear thick secretion revealing hydrocolpos.

MRI was recommended to further delineate the anatomy and pathology but the patient's family refused for MRI because of financial issues. The patient was planned for transvaginal evacuation of hematocolpos under intravenous anesthesia. On inspection, the right hemivagina was imperforated and was bulged out towards the introitus (Figure 2A). The imperforate vagina was punctured with a syringe, and a clear thick water- secretion was noticed which revealed the presence of hydrocolpos instead of hematocolpos as suggested by ultrasonography (Figure 2B).

After that vaginal septoplasty with marsupialization of vaginal septum was done. Her postoperative period was uneventful. On two weeks follow up, her symptoms of pain abdomen was improved and on examination vulva and vagina appeared normal. On per speculum examination, cervix was visualised with no any bulging or bleeding. Patient started to have a regular menstrual cycle of 28±2 days and have not developed any symptoms of recurrence.

## DISCUSSION

Herlyn-Werner-Wunderlich Syndrome (HWWS) was first reported by Purslow (1922) and Embrey (1950) and later elaborated by Herlyn, Werner and Wunderlich.^5^ HWWS is a combination of type III uterine anomaly and type Ia vaginal anomaly according to American society of reproductive medicine classification of mullerian duct anomaly along with ipsilateral renal agenesis.^3^ Though the actual cause behind failure of vertical and lateral fusion of mullerian duct and anomaly of wolffian duct is not known, some studies has suggested its association with genes related to renal agenesis like CHD1L, TRIM32, RET and WNT4.^2^

According to a case series by Gholoum et al. median age at presentation is 14 with symptom duration ranging from 0.5 to 12 months, while jong et al. has found two different age of presentation according to degree of hemivaginal obstruction as 12.86±1.84 years for complete and 20.68±7.43 for incomplete obstruction.^4,6^ Most of the patients are post pubertal and present shortly after menarche.^7^ In our case, patient presented at the age of 14 years before attending menarche with symptom duration of only 4 days.

As the patients attend menarche, menstrual blood starts collecting above obstructed hemivagina forming hematometrocolpos which results into multiple symptoms.^8^ So, patients mostly present with abdominal pain on the side of obstructed hemivagina, pelvic mass and dysmenorrhea.^2,4,7^ Some cases has also been reported where patient presented with acute urinary retention, septic shock and acute abdomen mimicking acute appendicitis.^9^ In our case, patient presented with pain over right flank since 4 days with hydrocolpos as a cause of symptom rather than hematocolpos. If the case is not diagnosed and treated on time, patients present with more serious complications like pyocolpos, pyosalpinx, peritonitis and tuboovarian abscess. All these complication ultimately lead to long term sequelae like endometriosis, adhesions, recurrent abortion and infertility.^3,7^ In our case, patient presented early with hydrocolpos without complications.

For the diagnosis, detailed clinical evaluation must be done at first followed by radiological investigations. USG is non invasive, fast and first investigation of choice which shows renal agenesis, two distinct uterine cavity and a cystic fluid collection in hemivaginal canal.^9,10^ MRI can be done further delineate the anatomy of uterus, vagina, kidney and to characterize the fluid collection. It can also detect associated complications like endometriosis, adhesions and pelvic inflammation.^3,7^ Laparoscopy though not done commonly is a gold standard procedure as it both helps in diagnosis and treatment.^3^ Han et al. has reported a case diagnosed in prenatal sonography and suggested for screening for HWWS in fetus with renal agenesis.^11^ In our case, patient was diagnosed in USG, MRI was recommended but was refused.

Excision of vaginal septum with drainage of hematocolpos is the treatment of choice, which can be done either transvaginal or hysteroscopic.^4,7,10^ In a case presented by Kim et al, hysteroscopic resection of vaginal septum with preservation of hymen integrity was done without using speculum or tenaculum.^10^ In another case of 12 years female with HWWS, hemihysterectomy of left side with removal of left tube was done as left side was found to be attretic during hysteroscopy.^7^ In a case series including 12 patients, surgical management with vaginal septectomy and drainage of hematocolpos or hematometrocolpos was done while one patient underwent salpingectomy for pyosalpinx.^4^ In our case, transvaginal drainage of collection revealed hydrocolpos which signifies absent menarche. Transvaginal drainage and vaginal septoplasty with septum marsupialization was done.

Patient has good prognosis in terms of sexual life and fertility after treatment though recurrent abortion can occur.^2^ Regular follow up with USG and renal function test is recommended for unilateral agenesis.^7^ In a case series including 12 patients, 11 patients became asymptomatic while 1 patient had irregular menstruation on follow up.^4^ In our case, patient was symptomatically better on two weeks follow up with examination findings suggesting no any complications. Patient started to have regular menstrual cycle.

## CONCLUSIONS

Early diagnosis and timely surgical intervention in managing Herlyn-Werner-Wunderlich Syndrome is crucial. The successful outcome, marked by symptomatic improvement and the initiation of regular menstrual cycles, highlights the effectiveness of transvaginal drainage of hydrocolpos and vaginal septoplasty. The case emphasizes the significance of a high index of suspicion in young females presenting with symptoms related to HWWS and contributes valuable insights to the existing literature on this rare congenital anomaly.
